# Low Incidence of Cancer Recorded in the Galapagos Archipelago

**DOI:** 10.1002/cnr2.70028

**Published:** 2024-12-26

**Authors:** Stefano Nicolas Chisesi, Massimo Rugge, Giulia Raffaella Galli, Martina Manni, Veronica Mishell Luzuriaga Delgado, Teodoro Chisesi, Juan Carlos Ruiz, Rina Mariuxi Quinto Briones, Luisa Siculella, Stefano Guzzinati, Manuel Zorzi, Massimo Federico, Anna Iannone

**Affiliations:** ^1^ Angela Serra Association for Cancer Research Pierto Baquerizo Moreno, San Cristobal, Galapagos Ecuador; ^2^ Department of Medicine (DIMED), Surgical Pathology and Cytopathology Unit University of Padova Padova Italy; ^3^ Veneto Cancer Registry Azienda Zero of the Veneto Region Padova Italy; ^4^ Department of Biological and Environmental Sciences and Technologies University of Salento Lecce Italy; ^5^ Department of Medical and Surgical Sciences for Children and Adults University of Modena and Reggio Emilia Modena Italy; ^6^ University of Specialties Espíritu Santo Guayaquil Ecuador; ^7^ Departamento de Campaña Preventiva of Guayaquil Guayaquil Ecuador; ^8^ Surgical, Medical and Dental Department of Morphological Sciences Related to Transplant, Oncology and Regenerative Medicine University of Modena and Reggio Emilia Modena Italy

**Keywords:** cancer incidence, cancer registration, cancer registry, Galapagos Islands

## Abstract

**Background:**

Cancer incidence in the Galapagos archipelago is unknown.

**Aim:**

In 2021, a task force including Ecuadorian and Italian researchers was established to estimate cancer incidence among the 25 244 Galapagos residents.

**Methods:**

Registration covered all malignancies, including malignant melanoma and non‐melanoma skin cancers; case recording was based on the International Classification of Diseases for Oncology. The data collection involved an active search across all relevant health institutions on the islands and the mainland. Mortality data were obtained from the Ecuador national mortality registry.

**Results:**

From January 2013 and December 2019, 174 new cancer cases were recorded, including 134 malignancies (M:F = 58:76) and 40 non‐melanoma skin cancers. The mean age at diagnosis was 48 years for males and 56 years for females. Prostate, gastric, and melanocytic malignancies were most incident among males; breast, thyroid, and cervical cancers prevailed in females. The age‐standardized incidence rates (ASR) were 80.39 for males and 99.24 for females with a mortality‐to‐incidence ratio 0.43. These ASRs were significantly lower than those reported in continental Ecuador and other South American countries.

**Conclusions:**

This pilot cancer registration initiative in the Galapagos record a low incidence of malignancies and requires validation with temporal expansion of cancer registration. The environmental etiology of some of the most common cancers warrants strategic primary and secondary prevention efforts.

## 1 Introduction

Based on the Ecuadorian National Institute of Statistics and Census (INEC), the resident population of the Galapagos in 2015 amounted to 25 244 individuals, almost all of Ecuadorean ancestry [1, 2]. Several studies have addressed the anthropology, geology, flora, and fauna of the Galapagos Archipelago. However, cancer epidemiology within this isolated community remains largely unexplored, with prior data limited to cancer‐related mortality reports.

Between 2013 and 2019, in the Galapagos population, the INEC reported 50 cancer‐related deaths, corresponding to an age‐standardized rate (ASR) of 35.98 cases per 100 000 for males and 21.81 for females [2]. These findings contrast sharply with the higher ASRs reported in Quito, the Ecuador capital, where cancer‐related mortality rates were 123.6 for males and 107.9 for females during the same period [3].

Currently, no population‐based data on cancer incidence in the Galapagos is available. This pilot study seeks to lay the groundwork for understanding cancer incidence in the Galapagos Province. The gathered information is essential for developing cancer prevention strategies and care programs tailored to local health priorities.

In December 2021, the University of Specialties Espíritu Santo (UEES Samborondón, Ecuador), the Italian Universities of Modena and Reggio Emilia, Padova, and Salento, and the Angela Serra Association for Cancer Research (Modena, Italy) established an international task force to explore cancer incidence among Galapagos residents. The study was based on a formal cooperation agreement with the Archipelago's authorities. This report concerns the results on cancer incidence in the Galapagos Archipelago between January 1, 2013 and December 31, 2019.

## 2 Methods

### 2.1 Ecuador's Territory, Administration, and Healthcare Context

Ecuador is a South American country located in the western region, bordering the Pacific Ocean. It includes four geographical regions: the Amazon, Highlands, Coast, and the Galapagos archipelago. These regions are further divided into 24 administrative provinces, which are then subdivided into 224 cantons. The total area of Ecuador is 283 561 km^2^, with an overall population of 18 000 000 as of 2022.

There are public primary health facilities on mainland Ecuador and its islands known as “Centros de Salud” focused on providing healthcare to small communities and rural areas. Additionally, “basic” second‐level hospitals are committed to admitting patients with non‐critical (acute or chronic) diseases for short‐term care. This healthcare local network also includes the recently established (year 2022) SOLCA cancer center (Sociedad de Lucha Contra el Cáncer del Ecuador) located in Santa Cruz Island. Particularly for cancer diseases, the archipelago‐settled health‐system collaborates with mainland third‐level hospitals equipped to handle all health diagnostic and therapeutic specialties and sub‐specialties, including oncology [4].

Cancer screening in the Galapagos is currently limited, with only voluntary cervical cancer secondary prevention programs in place, and no official data on population adherence.

### 2.2 Strategies and Procedures in Cancer Recording

This population‐based study covered all 25 244 residents of the Archipelago province, accounting for 0.16% of the population of Ecuador. The age distribution was as follows: 0–14 years: 27.7%, 15–29 years: 24.3%, 30–64 years: 43.4%, and ≥ 65 years: 4.1%.

A multidisciplinary team of seven local cancer registrars (G.R.G., M.M., S.N.C., V.M.L.D., J.C.R., R.M.Q.B., and L.S.), supervised by two researchers trained at the Veneto Cancer Registry (Padova, Italy) and Guayaquil Cancer Registry (G.R.G. and S.N.C.; Ecuador), conducted the data collection [5, 6].

Registration covered all malignancies, including cutaneous malignant melanoma (CMM) and non‐melanoma skin cancers. Cases were classified according to the International Classification of Diseases for Oncology [7, 8, 9]. Cancer recording was based on an active search in all Galapagos health institutions potentially involved in diagnosing cancer (Hospital Basico Republica del Ecuador in Santa Cruz, Hospital Oskar Jandl in San Cristobal, islands' *Centros de Salud*). A specific resident‐code identifies the Galapagos residents, excluding from cancer registration non‐resident subjects. To validate cancer cases with unavailable formal reporting at the archipelago level, appropriate searches were also conducted in mainland Ecuadorian oncology third‐level health care institutions.

Cancer registration was based on: (a) microscopic diagnosis, including histology and/or cytology of primary or metastatic cancer; (b) imaging, regardless of the technique used; (c) clinical information, often triggering more targeted investigations. Death certificates were used when explicitly mentioning cancer pathology or in support of already recorded cancer cases. Coding procedures (including the basis for, and the date of diagnosis) were adopted in accordance with current international guidelines [7, 8, 9, 10]. Age standardization rates (ASR) of cancer incidence were obtained using the direct method, based on the world standard population [11].

The mortality‐to‐incidence ratio was determined by dividing the number of cancer‐related deaths by the number of newly diagnosed cases within the study period [2].

Cancer registration relied on internally developed data entry methods and statistical software (VCR and GCR). Specific procedures were implemented to check data reliability, ensuring consistency with the International Agency for Research on Cancer data‐checking systems [10].

Ethical approval for the study was granted by the Municipal Ethics Authority of San Cristobal and the Ethics Committee for Research on Human Beings of the University of Specialties Espíritu Santo (CEISH‐UEES; protocol number 2022‐001C).

## 3 Results

Between January 1, 2013 and December 31, 2019, a total of 174 incident cases of cancer were recorded among Galapagos residents, including 134 malignancies (males [M]: 58; females [F]: 76) and 40 non‐melanoma skin cancers (Table 1). The most valid basis of diagnosis for each cancer site, distinguished by sex, is reported in Table S1a–c. Overall, 83.5% of all cancers were documented microscopically (histology and/or cytology on primary malignancy and/or metastasis), 14.2% based on clinical history, and 2.3% on imaging.

**TABLE 1:** Years 2013–2019: Crude and age‐standardized incidence rates (ASR world) × 100 000 in the Galapagos Islands, by site and sex.

Primary cancer site	Male				Female				Total				ICD10
	Case number	Frequency (%)	Crude rate	ASR world	Case number	Frequency (%)	Crude rate	ASR world	Case number	Frequency (%)	Crude rate	ASR world	
			Per 100 000				Per 100 000				Per 100 000		
Lip	1	1.2	1.11	1.18	1	1.1	1.16	0.77	2	1.1	1.13	0.99	*C00*
Tongue	0	0.0	0	0.0	0	0.0	0	0.0	0	0.0	0	0.0	*C01‐02*
Mouth	1	1.2	1.11	1.00	1	1.1	1.16	1.13	2	1.1	1.13	1.06	*C03‐06*
Salivary glands	0	0.0	0	0.0	0	0.0	0	0.0	0	0.0	0	0.0	*C07‐08*
Tonsil	0	0.0	0	0.0	0	0.0	0	0.0	0	0.0	0	0.0	*C09*
Other oropharynx	0	0.0	0	0.0	0	0.0	0	0.0	0	0.0	0	0.0	*C10*
Nasopharynx	0	0.0	0	0.0	0	0.0	0	0.0	0	0.0	0	0.0	*C11*
Hypopharynx	0	0.0	0	0.0	0	0.0	0	0.0	0	0.0	0	0.0	*C12‐13*
Pharynx unspecified	0	0.0	0	0.0	0	0.0	0	0.0	0	0.0	0	0.0	*C14*
Esophagus	1	1.2	1.11	1.00	1	1.1	1.16	2.00	2	1.1	1.13	1.42	*C15*
Stomach	5	6.1	5.53	6.58	6	6.5	6.96	9.43	11	6.3	6.22	7.87	*C16*
Small intestine	1	1.2	1.11	1.96	0	0.0	0	0.0	1	0.6	0.57	1.04	*C17*
Colon	3	3.7	3.32	4.60	1	1.1	1.16	2.41	4	2.3	2.26	3.49	*C18*
Rectum	1	1.2	1.11	1.61	0	0.0	0	0.0	1	0.6	0.57	0.89	*C19‐20*
Anus	1	1.2	1.11	1.96	0	0.0	0	0.0	1	0.6	0.57	1.04	*C21*
Liver	1	1.2	1.11	1.61	2	2.2	2.32	2.91	3	1.7	1.70	2.27	*C22*
Gallbladder etc.	0	0.0	0	0.0	0	0.0	0	0.0	0	0.0	0	0.0	*C23‐24*
Pancreas	1	1.2	1.11	1.53	0	0.0	0	0.0	1	0.6	0.57	0.85	*C25*
Nose, sinuses, etc.	0	0.0	0	0.0	0	0.0	0	0.0	0	0.0	0	0.0	*C30‐31*
Larynx	1	1.2	1.11	1.61	0	0.0	0	0.0	1	0.6	0.57	0.89	*C32*
Trachea, bronchus, lung	4	4.9	4.42	5.90	4	4.3	4.64	5.18	8	4.6	4.53	5.67	*C33‐34*
Other thoracic organs	0	0.0	0	0.0	0	0.0	0	0.0	0	0.0	0	0.0	*C37‐38*
Bone	4	4.9	4.42	5.68	0	0.0	0	0.0	4	2.3	1.70	2.37	*C40‐41*
Cutaneous melanoma	5	6.1	5.53	6.02	1	1.1	1.16	1.89	6	3.4	3.40	4.15	*C43*
Other skin	24	29.3	26.52	32.81	16	17.4	18.56	26.55	40	23.0	22.64	29.73	*C44*
Mesothelioma	0	0.0	0	0.0	0	0.0	0	0.0	0	0.0	0	0.0	*C45*
Kaposi sarcoma	1	1.2	1.11	1.11	0	0.0	0	0.0	1	0.6	0.57	0.58	*C46*
Connective and soft tissue	0	0.0	0	0.0	0	0.0	0	0.0	0	0.0	0	0.0	*C47 + C49*
Breast	0	0.0	0	0.0	19	20.7	22.04	24.19	19	10.9	10.75	11.49	*C50*
Vulva	0	0.0	0	0.0	1	1.1	1.16	1.10	1	0.6	0.57	0.54	*C51*
Vagina	0	0.0	0	0.0	0	0.0	0	0.0	0	0.0	0	0.0	*C52*
Cervix uteri	0	0.0	0	0.0	11	12.0	12.76	12.89	11	6.3	6.22	6.08	*C53*
Corpus uteri	0	0.0	0	0.0	3	3.3	3.48	5.22	3	1.7	1.70	2.37	*C54*
Uterus unspecified	0	0.0	0	0.0	0	0.0	0	0.0	0	0.0	0	0.0	*C55*
Ovary	0	0.0	0	0.0	6	6.5	6.96	6.97	6	3.4	3.40	3.24	*C56*
Other female genital organs	0	0.0	0	0.0	0	0.0	0	0.0	0	0.0	0	0.0	*C57*
Placenta	0	0.0	0	0.0	0	0.0	0	0.0	0	0.0	0	0.0	*C58*
Penis	0	0.0	0	0.0	0	0.0	0	0.0	0	0.0	0	0.0	*C60*
Prostate	9	11.0	9.95	14.34	0	0.0	0	0.0	9	5.2	5.09	7.89	*C61*
Testis	0	0.0	0	0.0	0	0.0	0	0.0	0	0.0	0	0.0	*C62*
Other male genital organs	1	1.2	1.11	1.18	0	0.0	0	0.0	1	0.6	0.57	0.60	*C63*
Kidney	3	3.7	3.32	3.84	0	0.0	0	0.0	3	1.7	1.70	2.08	*C64*
Renal pelvis	0	0.0	0	0.0	0	0.0	0	0.0	0	0.0	0	0.0	*C65*
Ureter	0	0.0	0	0.0	0	0.0	0	0.0	0	0.0	0	0.0	*C66*
Urinary bladder	0	0.0	0	0.0	1	1.1	1.16	1.22	1	0.6	0.57	0.58	*C67*
Other urinary organs	0	0.0	0	0.0	0	0.0	0	0.0	0	0.0	0	0.0	*C68*
Eye	0	0.0	0	0.0	0	0.0	0	0.0	0	0.0	0	0.0	*C69*
Brain. nervous system	2	2.4	2.21	1.96	0	0.0	0	0.0	2	1.1	1.13	0.86	*C70‐72*
Thyroid	2	2.4	2.21	1.75	11	12.0	12.76	12.89	13	7.5	7.36	6.98	*C73*
Adrenal gland	0	0.0	0	0.0	1	1.1	1.16	1.13	1	0.6	0.57	0.53	*C74*
Other endocrine	0	0.0	0	0.0	0	0.0	0	0.0	0	0.0	0	0.0	*C75*
Hodgkin lymphoma	0	0.0	0	0.0	2	2.2	2.32	2.00	2	1.1	1.13	0.97	*C81*
Non‐Hodgkin Lymphoma	3	3.7	3.32	4.39	0	0.0	0	0.0	3	1.7	1.70	2.45	*C82‐86.C96*
Immunoproliferative disease	0	0.0	0	0.0	0	0.0	0	0.0	0	0.0	0	0.0	*C88*
Multiple myeloma	1	1.2	1.11	1.78	2	2.2	2.32	3.23	3	1.7	1.70	2.58	*C90*
Lymphoid leukemia	3	3.7	3.32	4.78	1	1.1	1.16	1.59	4	2.3	2.26	3.27	*C91*
Myeloid leukemia	2	2.4	2.21	2.09	1	1.1	1.16	1.10	3	1.7	1.70	1.61	*C92‐94*
Leukemia unspecified	1	1.2	1.11	1.23	0	0.0	0	0.0	1	0.6	0.57	0.61	*C95*
Myeloproliferative disorders	0	0.0	0	0.0	0	0.0	0	0.0	0	0.0	0	0.0	*MPD*
Myelodysplastic syndromes	0	0.0	0	0.0	0	0.0	0	0.0	0	0.0	0	0.0	*MDS*
Other and unspecified	0	0.0	0	0.0	0	0.0	0	0.0	0	0.0	0	0.0	*O&U*
**All sites**	**82**	**100%**	**90.63**	**113.20**	**92**	**100%**	**106.70**	**125.79**	**174**	**100%**	**98.47**	**119.80**	***C00‐96***
**All sites except C44**	**58**		**64.10**	**80.39**	**76**		**88.14**	**99.24**	**134**		**75.83**	**90.06**	***C00‐96***

*Note:* C44 “non melanomatous skin cancers” include malignancies with different primary site and heterogeneous clinico‐biological profile; because of this peculiar profile, they are included among malignancies and also considered as separate entities.

Abbreviations: ASR, age‐standardized rate; ICD10, International Classification of Diseases.

The mean age at diagnosis was 56 years for males, and 48 for females. Patients' age distribution is shown in Figure S1.

The crude incidence rate was 64.10 for males and 88.14 for females, and the overall ASR was 90.06 per 100 000 population (M = 80.39; F = 99.24). Comparing the incidence data with the deaths reported by the INEC, the mortality‐to‐incidence ratio was 0.43 [2].

In males, malignancies most frequently involved the prostate (9 cases; ASR = 14.34), stomach (5 cases; ASR = 6.58), cutaneous malignant melanoma ([CMM] 5 cases; ASR = 6.02), the trachea/bronchus/lung (4 cases; ASR = 5.90), and bone (4 cases; ASR = 5.68) (Table 2). A very low incidence of colon cancer (3 cases; ASR = 4.59) was recorded.

**TABLE 2:** Years 2013–2019: The five most common malignancies in Galapagos (by sex and overall).

Males				Females				Overall malignancies			
The five most incident malignancies				The five most incident malignancies				The five most incident malignancies			
Primary site (ICD10)	Case #	Crude rate	ASR world	Primary site (ICD10)	Case #	Crude rate	ASR world	Primary site (ICD10)	Case #	Crude rate	ASR world
		Per 100 000				Per 100 000				Per 100 000	
Prostate (C61)	9	9.95	14.34	Breast (C50)	19	22.04	24.19	Breast (C50)	19	10.75	11.49
Stomach (C16)	5	5.53	6.58	Cervix uteri (C53)	11	12.76	12.89	Thyroid (C73)	13	7.36	6.98
CMM (C43)	5	5.53	6.02	Thyroid (C73)	11	12.76	12.89	Stomach (C16)	11	6.22	7.87
Respiratory (C33‐34)	4	4.42	5.90	Stomach (C16)	6	6.96	9.43	Cervix uteri (C53)	11	6.22	6.08
Bone (C40‐41)	4	4.42	5.68	Ovary (C56)	6	6.96	6.97	Prostate (C61)	9	5.09	7.89

*Note:* Cutaneous malignancies (others than cutaneous melanoma) are excluded because of their highly heterogeneous clinical and biological profiles. “Respiratory malignancies” include those originating from trachea, bronchus, lung (C33‐34).

Abbreviations: ASR, age‐standardized rate; CMM, Cutaneous malignant melanoma; ICD10, International Classification of Diseases‐ICD; 10th Revision code.

In females, the primary cancer most often involved the breast (19 cases; ASR = 24.19), cervix (11 cases; ASR = 12.89), thyroid (11 cases; ASR = 12.89), stomach (6 cases; ASR = 9.43) and ovary (6 cases; ASR = 6.97) (Table 2). The incidence of malignancies involving the colon (1 case; ASR = 2.40) or lung (3 cases; ASR = 4.08) was very low. Regardless of gender, Table 2 also reports the top five most common types of cancer in the archipelago.

## 4 Discussion

This pilot study examined the cancer incidence emerging from a newly established population‐based cancer registry in the Galapagos archipelago.

Based on the obtained findings, the overall incidence cancer rate among the residents of the Archipelago (ASR = 90.06) is significantly lower than that reported for continental Ecuador (ASR = 147) and all other South American countries, where ASR rates range from 257.8 (Uruguay) to 132.5 (Bolivia) (Table 3) [12, 13]. The ASR incidence rate recorded in Galapagos aligns with the low cancer‐related death rates reported in the archipelago population by the INEC from 2013 to 2019 [2].

**TABLE 3:** Estimated age‐standardized incidence rates (world) × 100 000 by sex in South American countries (2020).

Country	Male	Female	Total
Estimated age‐standardized incidence rates (world × 100 000)			
1. Uruguay	308.2	222.3	257.8
2. Argentina	222.7	209.1	212.4
3. Brazil	226.5	189.0	203.8
4. Paraguay	191.4	183.4	185.9
5. Colombia	178.9	178.6	177.5
6. Chile	199.3	155.8	173.5
7. Venezuela	170.4	173.0	170.1
8. Peru	161.0	180.5	169.4
9. Ecuador	140.7	160.0	149.7
10. Bolivia	116.2	150.2	132.5
**11. GALAPAGOS**	**80.39**	**99.24**	**90.06**

*Note:* Cancer incidence in all countries (lines 1–10) is reported as estimated national value of year 2020; line 11 refers to cancer registration, years 2013–2019.

In males, prostate and stomach cancers were the most incident malignancies in the Galapagos, with significantly lower ASR values compared to those reported in the mainland city of Quito (years 2011–2015: prostate cancer ASR = 14.3 in the Archipelago versus 61.8 in Quito; stomach cancer ASR = 6.5 in the Archipelago versus 19.6 in Quito; Table S2). Conversely, there was a different trend for CMM incidence, which was ranked third in the Galapagos (ASR = 6.0) and fifteenth in Quito (2011–2015 ASR = 4.7) [3]. This higher ASR incidence in the islands may be due to increased sun exposure of the islander residents, consistent with the recently reported increased mortality for melanocytic malignancies [14].

In the female residents of the Galapagos, the three most common primary malignancies were breast (ASR = 24.19), thyroid (ASR = 12.89), and uterine cervix (ASR = 12.89). However, these values were significantly lower than those recorded in the Ecuador continental capital of Quito (years 2011–2015) where the correspondent ASR values were 39.4, 40.9, and 17.7, respectively; Table S2 and were also much lower than in North America and Europe [11]. The high occurrence of “endocrine‐related” malignancies could possibly be linked to the increased rates of obesity recently reported in the Archipelago due to higher consumption of processed and ultra‐processed foods [15, 16].

The low incidence of lung and bladder malignancies, generally linked to environmental industrial pollution and tobacco smoking, is consistent with the trivial number of polluting industries in the Galapagos. There is little reliable information available, however, regarding the smoking habits of the resident population.

The results of this initial attempt at cancer registration in the Galapagos require further validation; additional efforts are needed to minimize the risk of losing cancer cases. This priority necessitates increased local access to digital data recording, promotion of the digitalized health information network, and extension of the registration period. Meeting these requirements is crucial for any reliable reinterpretation of the current findings in both their clinical and biological context. Despite the limitations mentioned above, the current findings suggest that the incidence of cancer in the Galapagos population is lower than in continental Ecuador and other South American countries. When validated by extending the registration time and supported by digital cancer recording, this epidemiological trend suggests region‐specific protective conditions or a peculiar impact of environmental risk factors. Both hypotheses require targeted investigations [17, 18].

This report outlines the strengths and weaknesses of a newly established population‐based cancer registration. Significant shortcomings are due to the geographical location of the territory, the lack of a digitalized network for health data collection, and the overall challenging healthcare context. These conditions may result in unidentified or misclassified cancer cases. Additionally, there is a need to improve the reliability of death certificates, as highlighted in a recent epidemiological study conducted in continental Ecuador: “There are potential problems in the description of the geographic distribution of specific causes of mortality, due to differences in the quality of death certification among provinces, which could introduce an important attribution bias” [13].

The point of strength is represented by the preliminary results obtained in cancer registration in a geographically confined, previously unexplored population. This initiative, after all the expected operational difficulties, has been currently endorsed by the local administration, which represents a crucial step forward in the local empowerment of the registration initiative [19]. A thorough search for incident cancer cases, achieved by involving all local health institutions, and a strategy that broadly exploits any complementary sources of information could improve the reliability of the cancer incidence recorded in the islands. The present findings are supported by their consistency with data on cancer mortality reported by institutional reports.

In summary, the initial results from the cancer registration in Galapagos show lower cancer rates compared to Ecuador and other countries in South America, the United States, and Europe. Further efforts are needed to minimize the risk of missing cancer by implementing province‐based digitalized recording procedures. The incidence of highly lethal malignancies like gastric and uterine cervix cancer, caused by infectious agents such as *Helicobacter pylori* and HPV, warrants proactive evaluation of primary and secondary prevention strategies.

## Author Contributions

**Stefano Nicolas Chisesi:** investigation, software. **Massimo Rugge:** conceptualization, writing – original draft, supervision. **Giulia Raffaella Galli:** investigation, software. **Martina Manni:** investigation, writing – original draft, software, data curation. **Veronica Mishell Luzuriaga Delgado:** investigation. **Teodoro Chisesi:** methodology, writing – review and editing. **Juan Carlos Ruiz:** investigation. **Rina Mariuxi Quinto Briones:** investigation. **Luisa Siculella:** methodology, formal analysis. **Stefano Guzzinati:** formal analysis. **Manuel Zorzi:** writing – review and editing. **Massimo Federico:** conceptualization, resources, project administration, funding acquisition, writing – original draft, writing – review and editing, validation, supervision, visualization. **Anna Iannone:** conceptualization.

## Conflicts of Interest

The authors declare no conflicts of interest.

## Supporting information


Figure S1.

Table S1.

Table S2.

## Data Availability

The data that support the findings of this study are available from the corresponding author upon reasonable request.
