# Supplementary material for: Low Incidence of Cancer Recorded in the Galapagos Archipelago
Source: Cancer Rep (Hoboken). 2024 Dec 26;7(12):e70028. doi: 10.1002/cnr2.70028 (PMC11670741; doi:10.1002/cnr2.70028)
Supplement: Supplementary file 1 — Figure S1. [file CNR2-7-e70028-s003.pptx]

## Slide 1
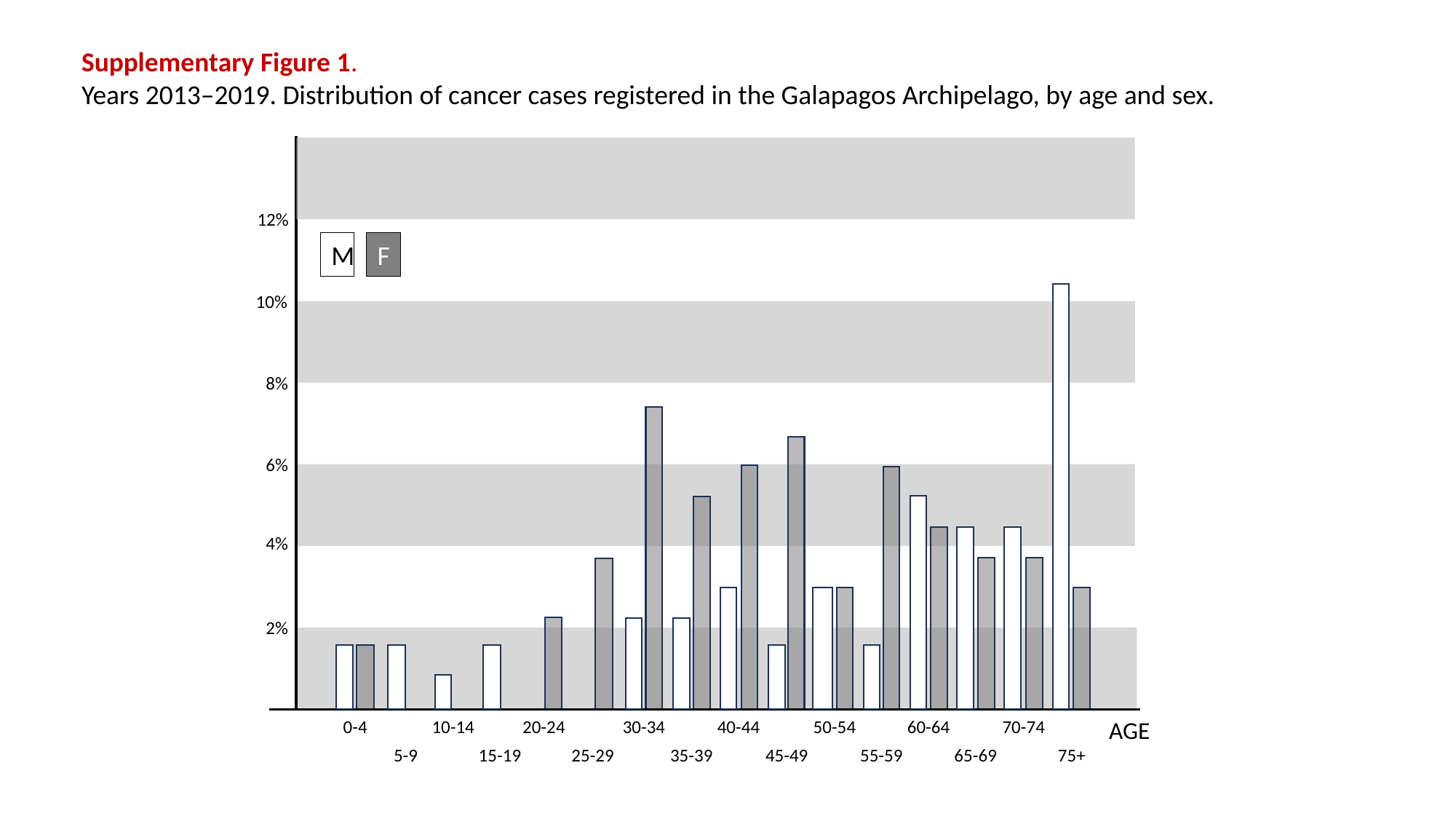

Supplementary Figure 1.
Years 2013–2019. Distribution of cancer cases registered in the Galapagos Archipelago, by age and sex.
12%
M
F
10%
8%
6%
4%
2%
0-4
10-14
20-24
30-34
40-44
50-54
60-64
70-74
AGE
5-9
15-19
25-29
35-39
45-49
55-59
65-69
75+
