# Supplementary material for: Low Incidence of Cancer Recorded in the Galapagos Archipelago
Source: Cancer Rep (Hoboken). 2024 Dec 26;7(12):e70028. doi: 10.1002/cnr2.70028 (PMC11670741; doi:10.1002/cnr2.70028)
Supplement: Supplementary file 2 — Table S1. [file CNR2-7-e70028-s002.docx]

**SUPPLEMETARY TABLE 1 (1a, 1b,1c)**

| **Supplementary Table 1a**. Confirmatory methods of diagnosis | | |
| --- | --- | --- |
| **Confirmatory Methods** | **Frequences** | **%** |
| Microscopy (Histology and/or Cytology, on Primary Tumor Histology on Metastasis, Level II Hematology) | 112 | 83,5 |
| Clinical History | 19 | 14,18 |
| Instrumental | 3 | 2,24 |
| **Total** | **134** | **100,0** |

| **Supplementary Table 1b.** Basis of diagnosis at each cancer site in males | | | |
| --- | --- | --- | --- |
| **Primary cancer Site** | **Clinical history** | **Histology on**  **primary tumor** | **Level II hematology** |
| Anus | 0 | 1 | 0 |
| Bone | 1 | 3 | 0 |
| Brain | 0 | 2 | 0 |
| Colon | 1 | 2 | 0 |
| Esophagus | 1 | 0 | 0 |
| Kaposi sarcoma | 0 | 1 | 0 |
| Kidney | 1 | 2 | 0 |
| Larynx | 0 | 1 | 0 |
| Leukaemia Unspecified | 0 | 1 | 0 |
| Lip | 1 | 0 | 0 |
| Liver | 0 | 1 | 0 |
| Lymphoid leukaemia | 0 | 2 | 1 |
| Melanoma of skin | 0 | 5 | 0 |
| Mouth | 1 | 0 | 0 |
| Multiple myeloma | 0 | 1 | 0 |
| Myeloid leukaemia | 0 | 2 | 0 |
| Non-hodgkin lymphoma | 0 | 3 | 0 |
| Other male genital organs | 1 | 0 | 0 |
| Pancreas | 0 | 1 | 0 |
| Prostate | 1 | 8 | 0 |
| Rectum | 1 | 0 | 0 |
| Small intestine | 0 | 1 | 0 |
| Stomach | 0 | 5 | 0 |
| Thyroid | 0 | 2 | 0 |
| Trachea, bronchi and lung | 3 | 4 | 0 |
| **Totale** | **11** | **46** | **1** |

| **Supplementary Table 1c.** Basis of diagnosis at each cancer site females | | | | | | |
| --- | --- | --- | --- | --- | --- | --- |
| **Primary cancer site** | **Clinical history** | **Cytology** | **Hematology II level** | **Histology**  **on metastasis** | **Histology on primary tumor** | **Instrumental** |
| Adrenal gland | 1 | 0 | 0 | 0 | 0 | 0 |
| Bladder | 1 | 0 | 0 | 0 | 0 | 0 |
| Breast | 2 | 0 | 0 | 0 | 14 | 3 |
| Cervix uteri | 2 | 4 | 0 | 0 | 5 | 0 |
| Colon | 0 | 0 | 0 | 0 | 1 | 0 |
| Corpus uteri | 0 | 0 | 0 | 0 | 3 | 0 |
| Esophagus | 1 | 0 | 0 | 0 | 0 | 0 |
| Hodgkin lymphoma | 0 | 0 | 0 | 0 | 2 | 0 |
| Lip | 1 | 0 | 0 | 0 | 0 | 0 |
| Liver | 0 | 0 | 0 | 0 | 2 | 0 |
| Lymphoid leukaemia | 0 | 0 | 0 | 0 | 1 | 0 |
| Melanoma of skin | 0 | 0 | 0 | 0 | 1 | 0 |
| Mouth | 0 | 0 | 0 | 0 | 1 | 0 |
| Multiple myeloma | 0 | 0 | 0 | 0 | 2 | 0 |
| Myeloid leukemia | 0 | 0 | 1 | 0 | 0 | 0 |
| Ovary | 0 | 0 | 0 | 1 | 5 | 0 |
| Stomach | 0 | 0 | 0 | 0 | 6 | 0 |
| Thyroid | 0 | 0 | 0 | 0 | 11 | 0 |
| Trachea, bronchi and lung | 0 | 0 | 0 | 0 | 4 | 0 |
| Vulva | 0 | 0 | 0 | 0 | 1 | 0 |
| **Totale** | **8** | **4** | **1** | **1** | **59** | **3** |
