# Supplementary material for: Low Incidence of Cancer Recorded in the Galapagos Archipelago
Source: Cancer Rep (Hoboken). 2024 Dec 26;7(12):e70028. doi: 10.1002/cnr2.70028 (PMC11670741; doi:10.1002/cnr2.70028)
Supplement: Supplementary file 3 — Table S2. [file CNR2-7-e70028-s001.docx]

**Supplementary Table 2.** Years 2011-2015: Crude and Age-standardized incidence rates (ASR World) x 100,000 in Quito, Ecuador by site and sex. The reported interval time (partially overlapping the time of the present study) is the only available official information for a large Ecuadorian population.

(ASR: age-standardized rate; ICD10: International Classification of Diseases (ICD) 10th Revision code). Reference: Sociedad de Lucha contra el Cáncer / Registro Nacional de Tumores. Cueva, P.; Yépez, J.; Tarupi, W. editores. 2019. Epidemiología del Cáncer en Quito 2011-2015. Quito. 16 ed.

|  | **MALE** | | | | **FEMALE** | | | | **ICD10** |
| --- | --- | --- | --- | --- | --- | --- | --- | --- | --- |
| PRIMARY CANCER SITE | CASE  NUMBER | Frequency (%) | Crude  Rate | ASR  World | CASE NUMBER | Frequency (%) | Crude  Rate | ASR World |  |
|  |  |  | per 100 000 | |  |  | per 100 000 | |  |
| Lip | 0 | 0.0 | 0 | 0 | 6 | 0.0 | 0.1 | 0.1 | *C00* |
| Tongue | 23 | 0.2 | 0.5 | 0.6 | 32 | 0.3 | 0.7 | 0.6 | *C01-02* |
| Mouth | 15 | 0.2 | 0.3 | 0.4 | 22 | 0.2 | 0.5 | 0.4 | *C03-06* |
| Salivary glands | 24 | 0.2 | 0.6 | 0.6 | 29 | 0.2 | 0.6 | 0.6 | *C07-08* |
| Tonsil | 6 | 0.1 | 0.1 | 0.2 | 1 | 0.0 | 0.0 | 0.0 | *C09* |
| Other oropharynx | 1 | 0.0 | 0.0 | 0.0 | 0 | 0.0 | 0 | 0 | *C10* |
| Nasopharynx | 4 | 0.0 | 0.1 | 0.1 | 3 | 0.0 | 0.1 | 0.1 | *C11* |
| Hypopharynx | 0 | 0.0 | 0 | 0 | 0 | 0.0 | 0 | 0 | *C12-13* |
| Pharynx unspecified | 2 | 0.0 | 0.0 | 0.1 | 0 | 0.0 | 0 | 0 | *C14* |
| Esophagus | 63 | 0.7 | 1.5 | 1.6 | 31 | 0.3 | 0.7 | 0.6 | *C15* |
| Stomach | 765 | 7.9 | 17.8 | 19.6 | 702 | 5.8 | 15.3 | 13.9 | *C16* |
| Small intestine | 56 | 0.6 | 1.3 | 1.5 | 54 | 0.4 | 1.2 | 1.1 | *C17* |
| Colon | 342 | 3.5 | 7.9 | 8.5 | 394 | 3.3 | 8.6 | 8 | *C18* |
| Rectum | 183 | 1.9 | 42 | 4.8 | 168 | 1.4 | 3.7 | 3.5 | *C19-20* |
| Anus | 16 | 0.2 | 0.4 | 0.4 | 56 | 0.5 | 1.2 | 1.2 | *C21* |
| Liver | 225 | 2.3 | 5.2 | 5.9 | 239 | 2.0 | 5.2 | 4.8 | *C22* |
| Gallbladder etc. | 163 | 1.7 | 3.8 | 4.3 | 268 | 2.2 | 5.8 | 5.5 | *C23-24* |
| Pancreas | 126 | 1.3 | 2.9 | 3.4 | 198 | 1.6 | 4.3 | 3.9 | *C25* |
| Other/ ill def. digestive organs | 3 | 0.0 | 0.1 | 0.1 | 3 | 0.0 | 0.1 | 0.1 | *C26* |
| Nose, sinuses, etc. | 29 | 0.3 | 0.7 | 0.7 | 18 | 0.1 | 0.4 | 0.4 | *C30-31* |
| Larynx | 62 | 0.6 | 1.4 | 1.7 | 12 | 0.1 | 0.3 | 0.3 | *C32* |
| Trachea, bronchus, lung | 290 | 3.0 | 6.7 | 7.4 | 311 | 2.6 | 6.8 | 6.4 | *C33-34* |
| Other thoracic organs | 14 | 0.1 | 0.3 | 0.3 | 10 | 0.1 | 0.2 | 0.2 | *C37-38* |
| Bone | 74 | 0.8 | 1.7 | 1.7 | 73 | 0.6 | 1.6 | 1.6 | *C40-41* |
| Cutaneous melanoma | 177 | 1.8 | 4.1 | 4.4 | 176 | 1.5 | 3.8 | 3.6 | *C43* |
| Other skin | 1622 | 16.8 | 37.7 | 41.1 | 1828 | 15.2 | 39.9 | 36.7 | *C44* |
| Mesothelioma | 14 | 0.1 | 0.3 | 0.4 | 7 | 0.1 | 0.2 | 0.1 | *C45* |
| Kaposi sarcoma | 51 | 0.5 | 1.2 | 1.2 | 8 | 0.1 | 0.2 | 0.2 | *C46* |
| Connective and soft tissue | 162 | 1.7 | 3.8 | 4 | 128 | 1.1 | 2.8 | 2.7 | *C47+C49* |
| Retroperitoneum and peritoneum | 34 | 0.4 | 0.8 | 0.9 | 43 | 0.4 | 0.9 | 0.9 | *C48* |
| Breast | 23 | 0.2 | 0.8 | 0.9 | 1819 | 15.1 | 39.7 | 39.4 | *C50* |
| Vulva | 0 | 0.0 | 0.5 | 0.6 | 25 | 0.2 | 0.5 | 0.5 | *C51* |
| Vagina | 0 | 0.0 | 0 | 0 | 26 | 0.2 | 0.6 | 0.6 | *C52* |
| Cervix uteri | 0 | 0.0 | 0 | 0 | 855 | 7.1 | 18.7 | 17.7 | *C53* |
| Corpus uteri | 0 | 0.0 | 0 | 0 | 284 | 2.4 | 6.2 |  | *C54* |
| Uterus unspecified | 0 | 0.0 | 0 | 0 | 64 | 0.5 | 1.4 | 6.2 | *C55* |
| Ovary | 0 | 0.0 | 0 | 0 | 384 | 3.2 | 8.4 | 8.1 | *C56* |
| Other female genital organs | 0 | 0.0 | 0 | 0 | 2 | 0.0 | 0.0 | 0.0 | *C57* |
| Placenta | 0 | 0.0 | 0 | 0 | 7 | 0.1 | 0.2 | 0.1 | *C58* |
| Penis | 46 | 0.5 | 1.1 | 1.1 | 0 | 0.0 | 0 | 0 | *C60* |
| Prostate | 2297 | 23.8 | 53.3 | 61.8 | 0 | 0.0 | 0 | 0 | *C61* |
| Testis | 265 | 2.7 | 6.2 | 5.6 | 0 | 0.0 | 0 | 0 | *C62* |
| Other male genital organs | 4 | 0.0 | 0.1 | 0.1 | 0 | 0.0 | 0 | 0 | *C63* |
| Kidney | 219 | 2.3 | 5.1 | 5.8 | 152 | 1.3 | 3.3 | 3.4 | *C64* |
| Renal pelvis | 3 | 0.0 | 0.1 | 0.1 | 1 | 0.0 | 0.0 | 0.0 | *C65* |
| Ureter | 1 | 0.0 | 0.0 | 0.0 | 1 | 0.0 | 0.0 | 0.0 | *C66* |
| Urinary bladder | 239 | 2.5 | 5.5 | 6.2 | 101 | 0.8 | 2.2 | 1.9 | *C67* |
| Other urinary organs | 4 | 0.0 | 0.1 | 0.1 | 1 | 0.0 | 0.0 | 0.0 | *C68* |
| Eye | 26 | 0.3 | 0.6 | 0.7 | 33 | 0.3 | 0.7 | 0.7 | *C69* |
| Brain. nervous system | 238 | 2.5 | 5.5 | 5.7 | 227 | 1.9 | 5 | 5 | *C70-72* |
| Thyroid | 338 | 3.5 | 7.8 | 8.2 | 1975 | 16.4 | 43.1 | 40.9 | *C73* |
| Adrenal gland | 7 | 0.1 | 0.2 | 0.2 | 3 | 0.0 | 0.1 | 0.1 | *C74* |
| Other endocrine | 14 | 0.1 | 0.3 | 0.3 | 5 | 0.0 | 0.1 | 0.1 | *C75* |
| Hodgkin lymphoma | 63 | 0.7 | 1.5 | 1.4 | 41 | 0.3 | 0.9 | 0.8 | *C81* |
| Non-Hodgkin Lymphoma | 603 | 6.2 | 14 | 14.9 | 548 | 4.5 | 12 | 11.5 | *C82-86.C96* |
| Immunoproliferative disease | 15 | 0.2 | 0.3 | 0.4 | 7 | 0.1 | 0.2 | 0.1 | *C88* |
| Multiple myeloma | 129 | 1.3 | 3 | 3.4 | 107 | 0.9 | 2.3 | 2.3 | *C90* |
| Lymphoid leukemia | 218 | 2.3 | 5.1 | 5.4 | 154 | 1.3 | 3.4 | 3.6 | *C91* |
| Myeloid leukemia | 187 | 1.9 | 4.3 | 4.5 | 137 | 1.1 | 2.9 | 2.8 | *C92-94* |
| Leukemia unspecified | 21 | 0.2 | 0.5 | 0.5 | 49 | 0.4 | 1.1 | 1 | *C95* |
| Myeloproliferative disorders | 19 | 0.2 | 0.4 | 0.5 | 29 | 0.2 | 0.7 | 0.9 | *MPD* |
| Myelodysplastic syndromes | 22 | 0.2 | 0.5 | 0.6 | 33 | 0.3 | 0.7 | 0.6 | *MDS* |
| Other and unspecified | 118 | 1.2 | 2.8 | 2.9 | 176 | 1.5 | 3.8 | 3.7 | *O&U* |
| **All sites** | **9665** | **100%** | **224.4** | **246.3** | **12066** | **100%** | **263.3** | **251** | ***C00-96*** |
| **All sites except C44** | **8043** |  | **186.7** | **205.2** | **10238** |  | **223.4** | **214.4** | ***C00-96*** |
